# Benefits and Difficulties of Implementing Family-Farming Food Purchases in the Brazilian National School Feeding Program

**DOI:** 10.3389/ijph.2024.1605870

**Published:** 2024-04-12

**Authors:** Mónica Isabel Eduardo Domingos Tuliende, Suellen Secchi Martinelli, Panmela Soares, Rafaela Karen Fabri, Vitória Uliana Bianchini, Suzi Barletto Cavalli

**Affiliations:** ^1^ Nutrition Postgraduate Programme, Federal University of Santa Catarina, Florianópolis, Brazil; ^2^ José Eduardo dos Santos University, Huambo, Angola; ^3^ Nutrition Department, Federal University of Santa Catarina, Florianópolis, Brazil; ^4^ Department of Community Nursing, Preventive Medicine and Public Health and History of Science, University of Alicante, Alicante, Spain; ^5^ Institute of Nutrition, Rio de Janeiro State University, Rio de Janeiro, Brazil

**Keywords:** schools, food supply, sustainable development, public policy, agriculture

## Abstract

**Objectives:** To explore the opinions of Brazilian National School Feeding Program (NSFP) nutritionists concerning the benefits and difficulties of implementing family-farming food purchases for the school feeding program.

**Methods:** Exploratory and descriptive qualitative study conducted through the analysis of inductive content of open interviews carried out with technically responsible nutritionists of the School Feeding Program of 21 municipalities in Southern Brazil.

**Results:** The qualitative analysis of the interviews resulted in 17 codes grouped into four categories that show the opinion of nutritionists on the benefits and difficulties of purchasing family-farming food: 1. increasing the visibility of rural areas and 2. improving the quality of food provided in school meals; 3. low product availability and 4. limited infrastructure for production and delivery.

**Conclusion:** According to nutritionists, purchasing family-farming food in NSFP can increase the supply of healthy food in schools and stimulate rural development. However, efforts are needed to adjust institutional food demands for local food production and improve infrastructure for food production and distribution.

## Introduction

There is a growing awareness of the power of institutional food services to promote sustainable food production and consumption through healthy food procurement strategies [1, 2]. The direct purchase policy of family-farming food in the School Feeding Program can contribute to developing more sustainable agrifood systems by reducing poverty and social inequality [3, 4], improving school meal quality [5–7], encouraging the incorporation of regional foods in school menus [8], diversifying family farm income sources [9] and increasing food security and nutrition [10–12].

The Brazilian National School Feeding Program (NSFP) provides school food and food education actions to all primary education students of the public education network. It provides meals to 41.5 million students during 200 school days with public funds [13]. In 2009, when the acquisition of family-farming products became mandatory in NSFP, the interaction between school food managers, family farms, and farmers’ organizations strengthened. Nutritionists have had to learn more about local production systems to incorporate locally grown foods in school menus [14, 15].

Nutritionists are NSFP technical managers responsible for planning school menus and monitoring meal preparation [16]. Thus, they can support local agricultural production, family-farming, and the production of organic or agroecological foods [14]. There is evidence that nutritionists are crucial players in implementing the purchase of family-farming food [17]. A study conducted in Rio de Janeiro suggests that municipalities with insufficient nutritionists struggled more in complying with the School Feeding Program regulations [18].

Studies showed that family-farming food purchased by public institutions results in improved meal quality by increasing the availability and variety of fruits, vegetables, and regional foods [6, 7, 19, 20]. However, several difficulties have been reported, including bureaucratic public procurement processes [7, 18, 19] and the limited production capacity of family farming to provide the required food products in sufficient amounts [19, 21, 22].

Although its benefits are recognized, the participation of family farming as a provider of school meals still needs to improve [19, 23]. Considering that the implementation of family-farming food purchase is conditioned to the planning of menus, knowing the opinion of SFP nutritionists about the benefits and difficulties of direct purchase can facilitate strategies that help these professionals include family-farming food in the SFP. Therefore, this study aims to explore the opinions of School Feeding Program (SFP) nutritionists concerning the benefits and difficulties of implementing the purchase of family farming food for school feeding in Southern Brazilian.

## Methods

An exploratory and descriptive qualitative study was conducted by analyzing the inductive content of open interviews with technically responsible nutritionists of the School Feeding Program of 21 municipalities in Southern Brazil.

We adopted cluster sampling by mesoregion of the states of Paraná, Santa Catarina, and Rio Grande do Sul, Brazil, to ensure the inclusion of nutritionists from municipalities with different socioeconomic, geographic, and cultural characteristics. Considering the specificities of the School Feeding Program planning process by number of students served, we opted to include nutritionists from municipalities with populations ranging from 20,000 to 70,000 inhabitants. The municipalities were randomly selected. Municipal Education Secretariats (NSFP Managing Unit in the state school system) were contacted by telephone and invited to participate in the study. All Municipal Education Secretariats that accepted to participate and had School Feeding Program nutritionists were included in the study. Two mesoregions in the state of Paraná were excluded from the study because they declined to participate or did not fulfill the inclusion criteria.

An interview guide was prepared by the research team in collaboration with nutrition experts and tested in a pilot study. The guide contained two open-ended questions to examine nutritionists’ opinions on the benefits and difficulties of purchasing family-farming food for school meals. Participants were assured that their answers would be confidential and never associated with their names. When necessary, the interviewer prompted participants to speak and probed for clarification or additional information.

Trained nutritionists conducted interviews between March and November 2015 during visits to the respondent’s workplace. Participants were informed about the study objectives and procedures and assured of their anonymity before the interviews. Participants signed an Informed Consent Form. The Research Ethics Committee of the Federal University of Santa Catarina approved this study under Protocol N° 1.002.956.

Interviews were recorded and transcribed *verbatim* with the participants’ consent. Qualitative content analysis was performed using NVivo 11 software. After transcripts were read repeatedly for familiarization, text fragments (words or phrases) with similar meanings were coded and grouped into categories. Two researchers engaged independently in data coding and categorization for high data reliability and then discussed their differences until a consensus was reached. We adopted open categorization, as the groups were not defined *a priori* but during data analysis.

## Results

Thirty-one nutritionists, technical managers of the School Feeding Program, participated in the study. This function was shared by more than one professional in eight municipalities, and interviews were collective. All respondents (*n* = 31) were females and had worked as School Feeding Program nutritionists for 8 years on average (range of 0.5–30 years). In all cases, the interviewed nutritionists planned school menus. The municipalities had an average of 34,000 inhabitants (range of 20,800 to 69,900) and 22 school units (range of 9–35). About 78,000 students were enrolled in the schools, and each school served, on average, 3,500 daily meals. The qualitative analysis of the interviews resulted in 17 codes that were grouped into four categories that show the opinion of nutritionists on the benefits and difficulties of implementing the purchase of family-farming food for the School Feeding Program (Table 1).

**TABLE 1:** Categories and codes used for the qualitative content analysis of interviews with School Feeding Program nutritionists about the benefits and difficulties of the implementation of purchase of family-farming food (Brazil, 2015).

	Benefit categories		Difficulty categories	
	Increased visibility for rural areas	Improved school meal quality	Low product availability	Lack of infrastructure for the production and delivery
Code	Incentives for rural producers	Higher availability of fresh foods	Low product variety	Low farmer motivation
	Commitment to food production	Higher availability of foods with low pesticide levels	Products available in insufficient amounts	Limited infrastructure for production, preparation, and delivery
	Increased interaction between rural actors	Incorporation of local, seasonal foods and regional dishes in the school menu	Seasonal variability in the food supply	Lack of municipal incentives
	Students become familiar with the food production chain	Increased food variety and increased offer of fruits and vegetables		High bureaucracy
		Higher nutritional quality		Lack of interaction between farmers

### Benefits of Purchasing Family-Farming Food for the School Feeding Program

The qualitative analysis resulted in two categories showing the opinion of the respondents on the benefits of purchasing food for the School Feeding Program: 1. increasing the visibility of rural areas and 2. improving the quality of food provided in school meals (Table 1).

#### Increased Visibility for Rural Areas

According to our results, drawing food producers and consumers closer positively affected farmers’ visibility. Family farmers’ commitment to producing quality food was strengthened by their involvement with the school setting. In the words of one of the respondents: “*The farmer also cares about the social aspect of School Feeding Programs, which is good. The farmer cares about quality, not just about selling. It is also for the children*.”

By increasing the visibility of family farming and its products, the School Feeding Program valued rural work and enhanced the pedagogical role of school meals in food education.

It brings the farmer closer to the student. It is that thing about food production. Children need to perceive food as food and be familiar with homemade biscuits, not just industrialized ones. Children have to know that milk and other things like that (...) come from somewhere, it is not just an industry. (Respondent 14)

#### Improved School Meal Quality

The respondents suggested that gathering production and consumption in schools favored the use of quality foods in school meals: Increasing the availability of fresh, seasonal foods that are part of the local gastronomic culture: “*Because foods are produced here, I can buy items that are part of the regional food culture, seasonal products” (Respondent 19). “The foods are harvested almost always on the day they are delivered” (Respondent 9)*.

Our results suggest the purchase of family-farming foods also contributed to increasing food variety and availability, which led to improved nutritional quality of meals: *“It increased nutritional quality. Because we currently have greater food variety. Nowadays, our goal is improving nutritional quality and variety” (Respondent 2).*

### Difficulties in Purchasing Family-Farming Food for the School Feeding Program

The qualitative analysis has given rise to two categories that show the opinions of the respondents on the difficulties of purchasing family-farming food for the School Feeding Program: 1. low product availability and 2. limited infrastructure for production and delivery (Table 1).

#### Low Product Availability

According to our results, adapting the school’s demand to the available foods produced in the region is a significant difficulty. *“We have to adapt to their products, and they have been producing the same foods for years” (Respondent 4).*

The nutritionists pointed out that the essential ingredients used in school meals are only sometimes produced in their region, hindering food purchases. The schools’ demands are often not met by local farms because of crop seasonality and low production volumes. *“Take onions and potatoes, for example. These are basic foods that we need but cannot find suppliers” (Respondent 9).*

#### Lack of Infrastructure for Food Production and Delivery

Analysis of the interviews suggests that food supply may be hampered by the lack of infrastructure of family farming for food production and delivery: *“They do not have a car, human resources or other things like that” (Respondent 6)*. This situation is aggravated by the complicated bureaucracy and lack of infrastructure in farmers’ organizations. The purchase of food by public institutions involves a series of bureaucratic procedures that sometimes hampers farmers’ participation: *“Because, in a contract, we have sales plans and many other things that they [the farmers] do not know how to do” (Respondent 21).*

In this sense, the lack of public technical assistance and municipal support was pointed out as a difficulty in purchasing food for the School Feeding Program: *“They [the farmers] say that there is a lack of technical assistance from the Secretary of Agriculture on how to maintain production, you know?” (Respondent 2).*

## Discussion

This study explored the opinions of School Feeding Program nutritionists concerning the benefits and difficulties of purchasing family-farming food for school feeding. The respondents affirmed that implementing family-farming food purchases has increased rural areas’ visibility and improved school meals’ quality. However, low product availability and limited infrastructure for production and distribution were identified as difficulties.

The findings agree with previous studies showing that the quality of school meals is enhanced by purchasing local family-farming food, mainly because of higher availability and variety of fresh foods [6, 7, 19, 20]. The supply of adequate food in schools can contribute to the acquisition of healthy eating habits, which can positively impact the population’s health in the long term [24].

School nutritionists believed that purchasing family-farming food increased farmer visibility, which, in the long term, may help valorize farmers’ work and reduce poverty and social inequalities in rural areas [3, 4]. This result indicates the program’s potential to strengthen fairer production and consumption forms, which is especially important considering that an essential part of people in a situation of food insecurity in the world live in rural areas [25].

As in previous studies [19, 21, 22], school Feeding Program nutritionists identified low product availability as a significant hindrance in purchasing family-farming food. Although purchases increased the availability and variety of foods in school meals, family farming could not provide a constant supply of widely used food items, such as potatoes, onions, and bananas, which may be because schools have a large daily food demand to meet all schoolchildren, or the menus need to consider production seasonality.

The purchase of family-farming food increases the complexity of school menu planning, requiring nutritionists to adapt to the reality of local food production. The success of the purchase depends on the characteristics of local farms. School menu planning must be articulated with agricultural production. Thus, part of the nutritionist’s tasks in the National School Feeding Program should include mapping the local production capacity, assessing the school’s demand, and bridging the gap between family farmers and school food managers [26–28]. School menu planning should only be carried out after assessing the local family farming characteristics.

School food managers, in turn, must recognize the particularities of purchasing family-farming food and adapt to this type of demand and supply, which requires participatory management. Education, agricultural, planning, procurement, and civil society sectors should connect and engage at the municipal, state, and federal levels [29]. Creating participatory spaces or debate forums for farmers, managers, school representatives, and the School Feeding Program board is crucial to reducing these operational hardships [7, 26]. School nutritionists are vital in coordinating exchanges between the several sectors purchasing family-farming foods [17, 18]. However, sustainability needs to be addressed as an essential aspect of the profession in the education and training of nutritionists [30]. Sustainable development should be considered in curriculum guidelines for this nutrition professional to strengthen healthy and sustainable food production chains.

The results highlight the difficulties of needing more technical assistance and insufficient infrastructure for producing and distributing food products. These results are similar to those found in other studies [19, 21]. Such issues can be overcome through farmers’ organizations and municipal aid. For instance, farmers’ cooperatives can hire personnel to deliver food products or share tasks among participants. The municipality can provide support by creating a center for delivering and distributing food to schools [31]. There is a need to expand public investment in food storage and distribution, which would increase the scale of family-farming food purchases. Farmers’ organizations can help farmers improve their technical capacity and mediate their relationship with public authorities [28]. Increased dialogue and technical assistance to family farming can minimize the difficulties in meeting the demands of schools [31].

We should consider that the respondents’ opinions may be influenced by their professional interests, as they were responsible for school menu planning. However, the nutritionists are crucial players in implementing the School Feeding Program, and knowing their opinion on purchasing family-farming food can develop strategies that favor its implementation. On the other hand, a neoliberal agenda was implemented in the country after data collection, impacting Brazilian public food policies. However, the results presented in the study allow us to know the opinion of nutritionists with experience in implementing family-farming food purchases. These results can be helpful for decision-makers when implementing public policies for acquiring family-farming food for the supply of food services of public institutions.

In conclusion, according to NSFP nutritionists, purchasing family-farming food can positively impact the provision of healthy, sustainable meals in schools. Food purchase from local family farming provides a greater supply and variety of fresh foods and increases the visibility of rural areas. However, efforts are needed to adjust institutional food demands to local food supply and enhance the food production and distribution infrastructure.

Our results suggest that school food managers need to recognize the particularities of purchasing family-farming food to overcome the barriers. To guarantee healthy and sustainable meals in schools, nutritionists should assess the capacity and characteristics of local food suppliers before planning school menus.
